# Venous Thromboembolism and Bleeding Risk in a Population with Obesity Hospitalized for Surgery and Receiving Enoxaparin for Thromboprophylaxis

**DOI:** 10.1007/s11695-026-08606-4

**Published:** 2026-05-18

**Authors:** Walter Ageno, Marc Carrier, Christine Stroh, Yasmina Djoudi, Mohamed Abdel-Moneim, Irfan Khan, Ekaterina Ponomareva

**Affiliations:** 1 Department of Medicine, University of Padua, Padua, Italy; 2 Ottawa Hospital Research Institute, University of Ottawa, Ottawa Hospital, Ottawa, Canada; 3 Department of Obesity and Metabolic Surgery and Proctology, SRH Hospital Gera, Gera, Germany; 4 Sanofi (France), Paris, France; 5 Sanofi (UAE), Dubai, United Arab Emirates; 6 University of Sharjah, Sharjah, United Arab Emirates; 7 Sanofi (United States), Bridgewater, USA; 8 Axtria Inc., Berkeley Heights, USA

**Keywords:** Venous thromboembolism, Hemorrhage, Obesity, Enoxaparin, Hospitalization, Prophylaxis

## Abstract

**Introduction:**

Obese patients hospitalized for surgery are at high risk of venous thromboembolism (VTE). The optimal dose and duration of thromboprophylaxis with low molecular weight heparin for these patients are uncertain.

**Aims:**

To assess the time-course, rates and risk factors for VTE and major bleeding (MB) in a population of surgical patients with obesity receiving pharmacological thromboprophylaxis with enoxaparin.

**Methods:**

Patients with body mass index (BMI) > 30 kg/m^2^ hospitalized with surgeries between 2010 and 2021 who received thromboprophylaxis with enoxaparin were selected from the US Optum database. Exclusion criteria were VTE, MB, or surgery in previous 90-days, and ongoing anticoagulant treatment or dual antiplatelet therapy. VTE and MB event rates over a 90-day follow-up post enoxaparin initiation were estimated via the Kaplan-Meier (KM) method. Risk factors associated with outcome events were identified via Cox proportional hazard models.

**Results:**

A total of 30,492 patients met selection criteria, 12,058 patients received the standard dose, with 18,300 receiving higher doses. KM event rates at 90-days for VTE and MB were 2.5% and 1.2%, respectively. The highest VTE rates were observed in patients hospitalized for thoracic surgery (4.9%). History of VTE was the strongest predictor of post-surgery VTE (HR 5.62, 95% CI 4.71–6.7) while history of MB was the strongest predictor of post-surgery bleeding (HR 2.62, 95% CI 1.29–5.32).

**Conclusions:**

The rates of VTE are non-negligible in surgical patients with obesity receiving thromboprophylaxis with enoxaparin. Individual risk stratification is warranted to identify optimal doses/duration of pharmacologic thromboprophylaxis.

**Supplementary Information:**

The online version contains supplementary material available at 10.1007/s11695-026-08606-4.

## Key Points

Patients with obesity hospitalized for surgery are at high risk of VTE and MB.

VTE rates estimated using claims data in this population were non-negligible.

History of VTE and thoracic surgery were associated with a higher risk for VTE 90 days post discharge.

Individual risk stratification is warranted to identify optimal dosage/duration.

**Supplementary Information:**

The online version contains supplementary material available at 10.1007/s11695-026-08606-4.

## Introduction

In 2024, it was estimated that more than one billion individuals (880 million adults and 159 million children) worldwide have obesity [1]. Obesity, defined as body mass index (BMI) ≥ 30 kg/m^2^, is a global epidemic linked to increased morbidity and mortality. It is also an independent risk factor for developing venous thromboembolic events (VTE) [2–4]. 

Patients undergoing major surgery already face a heightened risk of developing VTE, including deep-vein thrombosis and pulmonary embolism [5]. When obesity is present, this risk further increases, making patients with obesity more vulnerable to VTE and other adverse events during and after surgery. Several factors, including patient’s pre-existing conditions and the type of surgery performed, can influence the likelihood of both VTE and bleeding complications [6–8]. The risk of VTE remains particularly high within the first 30 days following hospital discharge [9–11]. In patients with obesity, this risk is estimated to be more than twice as high as in those without obesity and is further amplified when combined with additional thrombotic risk factors [12, 13]. 

Pharmacological thromboprophylaxis is generally warranted in patients undergoing major orthopedic or cancer surgery [5] However, RCTs and other studies provide very limited evidence regarding thromboprophylaxis in the population with obesity (BMI 40 kg/m^2^ or more) [14–18]. For example, the American Society for Metabolic and Bariatric Surgery (ASMBS) guidelines recommend metabolic and bariatric surgery for patients with BMI ≥ 30 kg/m^2^; however, the ASMBS does not provide specific guidelines for thromboprophylaxis following surgery in patients with obesity [19]. While it is recognized that the risk of VTE continues for extended periods of post-surgery hospital stay, there is no consensus regarding optimal VTE prophylaxis regimen in patients with obesity [4, 20, 21]. An evaluation of the risk for VTE and bleeding in this population is therefore critical for guiding VTE prophylaxis [22, 23]. 

In this study, we aimed to describe the dose and duration of thromboprophylaxis with the low molecular weight heparin, enoxaparin, prescribed in usual clinical practice for hospitalized surgical patients with obesity who underwent a surgical procedure, and to assess risk factors and time-course of event rates for postoperative VTE and major bleeding (MB).

## Methods

### Study Design

This study used a population drawn from the Optum Market Clarity database and represents individuals in the USA enrolled in health plans including private and Medicare Advantage, the latter being a private plan that contracts with Medicare and provides all of the benefits of Medicare part A (hospital insurance), Medicare part B (supplementary medical insurance, and often Medicare part D (privately sponsored prescription drug plans) [24]. The database comprises linked information from electronic health records, inpatient and outpatient claims, prescriptions, laboratory tests, and plan enrollment.

**Fig. 1:**
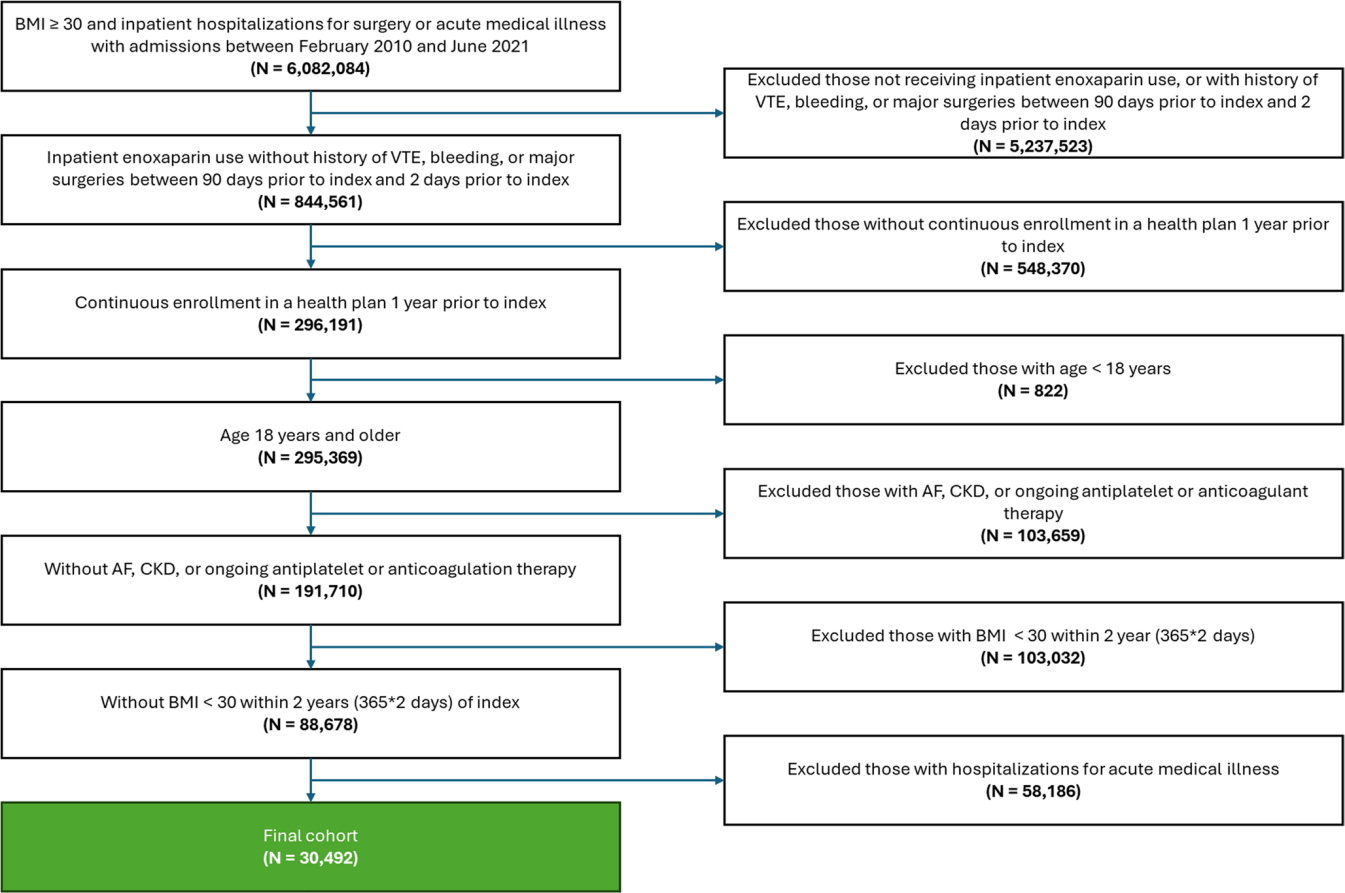
Selection of the study population from optum market clarity database. AF, atrial fibrillation; CKD, chronic kidney disease; VTE, venous thromboembolism

Patients hospitalized for elective or emergency surgery (abdominal/pelvic, orthopedic, and thoracic) between 28th February 2010 and 30th June 2021 and receiving enoxaparin thromboprophylaxis since admission were initially selected before the application of subsequent selection criteria (Fig. 1). If a patient had multiple qualifying hospitalizations, one was selected at random to facilitate the interpretation of the cohort as being comprised of unique patients [25]. However, patients in this analysis had at least one, and could have more than one surgery/type of surgery during their hospitalization. Additional inclusion criteria were hospitalization for a major surgery, age ≥ 18 years, ≥ 1-year of continuous enrollment in a health plan prior to index, and BMI ≥ 30 kg/m^2^. Patients were excluded if they had VTE or a MB event 90-days prior to index, major surgery within − 2 to − 90 days prior to index, ongoing antiplatelet/anticoagulant therapy (medication prescription that covered at least part of the period within − 2 to − 32 days prior to index), atrial fibrillation, chronic kidney disease (CKD) stages IV and V (identified through diagnoses codes or estimated glomerular filtration rate (eGFR) < 30 ml/min/1.73m^2^), or dialysis.

Index hospitalization was characterized by the patient’s reason for hospitalization, length of stay (LOS), and intensive care unit (ICU) stay. The index date was defined as the start of inpatient enoxaparin thromboprophylaxis. Clinical conditions were identified from medical diagnosis and procedure codes during the 1-year baseline period prior to index enoxaparin initiation. Ongoing medication use was ascertained from medication supply within 90 days of index; a 30-day grace period was allowed after the end of days of supply. Duration of thromboprophylaxis represented the time from first enoxaparin administration to either the last administration in hospital, or the last day of medication supply if the patient received post-discharge thromboprophylaxis. If there was an interruption of ≤ 2 days, the duration of thromboprophylaxis was assumed as continuous. Patients were categorized as receiving a usual or standard dose if the daily dose (that is, the overall amount of medicine received in one day) administered at index was ≤ 40 mg, while those who received > 40 mg were categorized as receiving a higher dose.

The outcome of endpoint was VTE, characterized as a new onset event occurring in either the inpatient or outpatient settings, and the safety outcome was MB, characterized as a new onset event in the inpatient setting. The ascertainment of VTE and MB endpoints was based on an algorithm that utilized diagnosis codes along with information on the setting (inpatient or outpatient).

To determine the VTE endpoint, we first reviewed and included all ICD-9 and ICD-10 codes that are specific to acute pulmonary embolism or deep vein thrombosis (DVT), removing all DVT codes that did not explicitly mention deep veins or identified veins that are deep (e.g., femoral). Next, we also removed codes that explicitly mentioned chronic. This set was used for identification of VTE events in inpatient setting. From this set, we then retained only those codes that explicitly mentioned acute onset event or pulmonary embolism in the definition and used these codes in outpatient setting. Further explanation as well as the final list of codes (Table S1) can be found in the supplement.

When determining the MB endpoint, An algorithm was developed using the International Society on Thrombosis and Haemostasis (ISTH) criteria, employing symptomatic bleeding in a critical area or organ—such as intracranial, intraspinal, intraocular, retroperitoneal, intraarticular, pericardial, or intramuscular bleeding with associated compartment syndrome—as the primary proxy for major bleeding. Towards this end any code indicating bleeding in an area or organ (critical or non-critical) was considered as major bleed if it occurred in the primary position on a claim in an inpatient setting. A subset of these codes was then considered as major bleeding for inpatient non-primary position. Any code explicitly mentioning “chronic” was not considered for non-primary position, and conversely any code explicitly mentioning “acute” was retained for non-primary position. Compartment syndrome was considered as MB if hemorrhage or hematoma occurred during the same hospitalization. Other codes were considered non-major bleeds and were used for baseline characterization of history of non-major bleeding. Detailed explanation as well as the final list of codes (Table S2) can be found in the supplement.

### Statistical Analysis

The algorithm summarized in Fig. 1 was informed by prior studies and refined by coauthors to ensure specificity for identifying new onset VTE and MB events. Patients were followed for VTE and MB events for up to 90 days from index, with censoring criteria defined as death, disenrollment from the health plan, or the end of the 90-day follow-up period, whichever came first. Cumulative incidence of VTE and MB events were estimated via survival analysis (1 – Kaplan-Meier [KM]). Cox proportional hazards models were utilized to investigate the association of baseline conditions with the risk of VTE and MB. The reported set of risk factors represented those that were retained after application of backward variable selection process (with *p* < 0.05) in the Cox models for either VTE or MB using Python (version 3.9.3) statistical software.

## Results

### Study Population

A total of 30,492 patients were selected based on the study inclusion criteria (Fig. 1). Baseline characteristics for the surgery group stratified by enoxaparin dosage are depicted in Table 1, with stratification by surgery type available in Table S3. Most patients were females, accounting for 67.6% of the population. A quarter (24.4%) of the patients were aged 65 and over. Patients with a BMI > 40 kg/m2 comprised 41% of the group. Hypertension was the most prevalent comorbidity at 53.5%. The most used medications were anticoagulants (within 3 months of index) (35.5%), and angiotensin converting enzyme inhibitors (ACEi)/angiotensin receptor blockers (ARB) (27.9%).

**Table 1:** Baseline characteristics for patients with obesity undergoing surgery

	Enoxaparin Dose^a^		Total, *N* = 30,492
	Usual Dose ≤ 40mg per day, *N* = 12,**058**	High Dose >40mg per day, *N* = 18,**300**	
**Demographics**			
Female sex (%)	68.7	67.0	67.6
Age, years (%)			
18—39 years	17.6	15.2	16.12
40—64 years	59.4	59.5	59.49
65—75 years	16.4	18.8	17.88
>75 years	6.6	6.5	6.5
Index Hospitalization Characteristics			
Length of hospital stay (days), median (IQR)	3 (2–5)	3 (3–5)	3 (2–5)
Admission to enoxaparin start (days), median (IQR)	1 (0–1)	1 (0–1)	1 (0–1)
Enoxaparin starts to discharge (days), median (IQR)	3 (2–4)	3 (2–4)	3 (2–4)
Duration of prophylaxis with enoxaparin (days), median (IQR)	2 (2–3)	3 (2–8)	2 (2–4)
ICU/ CCU Stay, (%)	12.6	13.5	13.1
Patients with enoxaparin Rx post discharge, (%)	3.8	2.8	3.2
BMI kg/m^2^ (24 months prior to index), (%)			
30 to 34.9	37.7	33.3	35.0
35-39.9	23.9	24.1	24.1
>40	38.3	42.7	40.9
Clinical Conditions, (%)			
Stroke and CBVD			
Hemorrhagic stroke	0.1	0.2	0.1
Ischemic stroke	1.2	1.3	1.3
Unspecified stroke or CBVD without stroke	3.5	4.4	4.1
Thrombophilia	0.4	1.0	0.8
Severe varicosities^b^	1.8	2.1	2.0
History of cancer	12.5	14.5	13.7
Gastroduodenal ulcer	9.7	10.1	10.0
Lower limb paralysis	0.2	0.2	0.2
Central venous catheter	2.0	2.6	2.4
Heart failure	2.6	2.8	2.7
COPD	8.3	7.8	8.0
CKD stage III	11.1	10.3	10.6
CHD	9.8	10.0	10.0
Drug misuse disorder^c^	14.9	15.3	15.2
HIV infection	1.3	0.8	1.0
History of tobacco use	50.9	47.9	49.6
Diabetes	23.9	23.7	23.8
Immuno-hematologic conditions^d^	0.8	0.9	0.8
Peripheral vascular disease	5.1	4.5	4.7
Moderate/severe chronic liver disease	8.1	8.8	8.5
History of VTE	4.0	5.9	5.2
History of Bleeding			
Major bleeding	0.6	0.7	0.7
Nonmajor bleeding	14.2	12.8	13.4
Medication Use,^d^ (%)			
Anticoagulants^e^	16.5	47.9	35.5
ACEi/ ARB	26.5	28.8	27.9
Beta blockers	22.1	17.6	19.5
Calcium channel blockers (CCBs)	13.1	11.7	12.3
Statins	18.9	22.2	20.9
Antiplatelets^f^	1.2	1.0	1.1
Hormone replacement therapy	1.7	1.5	1.6

Abbreviations: *ACEi* angiotensin-converting enzyme inhibitors, *ARB* angiotensin II receptor blockers, *CBVD* cerebrovascular disease, *COPD* chronic obstructive pulmonary disease, *CKD* chronic kidney disease, *CHD* coronary heart disease, *HIV* human immunodeficiency virus, *CCBs* calcium channel blockers, *HR* hazard ratio, *VTE* venous thromboembolism

^a^ 134 out of 30,492 patients in the surgery group had missing dosage. ^b^ Varicose veins of lower extremities with inflammation, varicose veins of lower extremities with ulcer and inflammation, and varicose veins with both ulcer and inflammation (ICD-9 454.1 and 454.2, ICD-10 I83.1 and I83.2).^c^ Includes codes for nicotine dependence, opioid dependence, other psychoactive substance abuse, other illegal prescription drug abuse. ^d^ Includes anemia, cell aplasia, pancytopenia, bone marrow failure syndromes, agranulocytosis, genetic anomalies of leukocytes, severe combined immunodeficiency (SCID), Nezelof’s syndrome, Wiskott-Aldrich syndrome, Di George’s syndrome, acute graft-versus-host disease. ^e^ Within 33–90 days prior to index. ^f^ Within 33–90 days prior to index

### Dosage/Duration of Thromboprophylaxis in the High and Low Dose Populations

For the 12,058 patients administered the standard dose of enoxaparin, 37.7% had a BMI of 30–34.9 kg/m2, 23.9% had a BMI of 35–39.9 kg/m2, and 38.3% had a BMI of 40 + kg/m^2^. Comparatively, the percentage of each group for the 18,300 patients given higher doses was 33.3%, 24.1%, and 42.7%, respectively. Median duration of thromboprophylaxis with enoxaparin was higher for the high dose population compared to the usual dose population (3 (IQR 2–8) vs. 2 (IQR 2–3) days). When comparing the subgroups of patients who received the high dose vs. the standard dose of enoxaparin, surgical patients with BMI > 40 kg/m^2^ (42.7% vs. 38.3%), history of VTE (5.9% vs. 4.0%), history of cancer (14.5% vs. 12.5%), prior anticoagulant use (47.9% vs. 16.5%), and prior statin use (22.2% vs. 18.9%) received higher doses (Table 1).

### Overall Duration of Hospitalization and Thromboprophylaxis

The median duration of hospitalization once thromboprophylaxis started was 3 days (IQR, 2–4 days). The median duration of enoxaparin thromboprophylaxis was on average shorter than the inpatient stay − 2 days (IQR 2–4 days); 11% of patients received enoxaparin for at least 7 days, 4% received enoxaparin for at least 14 days, and only 1% of patients received enoxaparin for 30 days or more (Figure S1).

### Time-Course, Event Rates and Risk Factors for VTE and MB

**Fig. 2:**
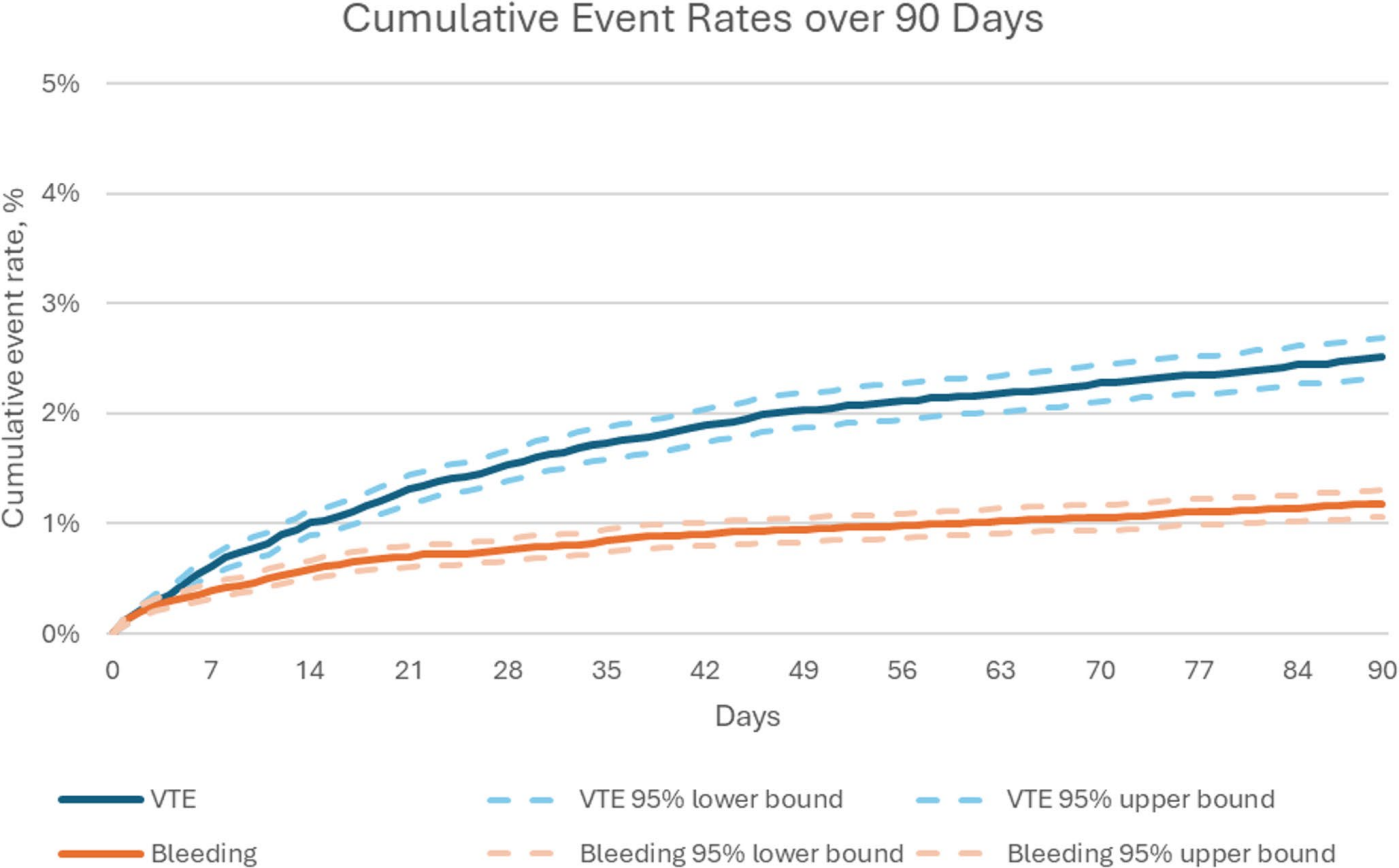
Kaplan-Meier Cumulative Incidence of VTE and MB event rates. Time zero indicates enoxaparin initiation. VTE, venous thromboembolism

The event rates for VTE and MB are summarized over time in Table S4. The cumulative VTE event rates were 0.6%, 1.6%, and 2.5% at 7, 30 and 90 days follow up and the MB event rates were 0.4%, 0.8%, and 1.2% respectively (Fig. 2, Table S4). The highest cumulative incidences of both VTE and MB were observed following thoracic surgeries (4.9% and 2.5% respectively) (Figure S2), while the lowest VTE and MB event rates were observed following abdominal/pelvic (1.7%) and orthopedic (0.7%) surgeries. Of these, 2.9% of VTE and 1% of MB for thoracic surgeries happened post-discharge, with 1.5% and 0.6% respectively for pelvic and orthopedic surgeries (Table S5).

Results from multivariable analysis for associations between baseline characteristics and outcomes are shown in Table 2. Hospitalization due to thoracic surgery was associated with higher rates of VTE (HR 2.61, 95% CI 2–3.4) as compared to hospitalizations with abdominal/pelvic surgeries. Patients > 75 years of age had a higher risk of both VTE and MB events, with HR 1.87 (95% CI 1.3–2.69) and HR 1.98 (95% CI 1.25–3.14), respectively. Prior history of VTE (HR 5.62, 95% CI 4.71–6.7) was a significant risk factor for VTE in the follow up. A history of cancer was similarly a significant risk factor for VTE (HR 1.6, 95% CI 1.32–1.93). History of major bleeding (HR 2.62, 95% CI 1.29–5.32), and non-major (HR 2.45, 95% CI 1.92–3.12) were most predictive of MB events in the follow-up period.

**Table 2:** Patient Characteristics at Index Associated with the Occurrence of VTE and Major Bleeding During 1-90-days Post Index: Cox Proportional Hazards Model

Abdominal/ Pelvic	*N* (%)	VTE HR (95% CI)	MB HR (95% CI)
	17,544 (57.5)	Reference	Reference
Orthopedic	11,582 (38.0)	1.86 (1.56–2.21)	0.5 (0.38–0.66)
Thoracic	1,749 (5.7)	2.61 (2–3.4)	1.39 (0.97–1.98)
Age, years			
18–39	4,915 (16.1)	Reference	Reference
40–64	18,141 (59.5)	1.3 (0.99–1.72)	0.73 (0.53–0.99)
65–75	5,453 (17.9)	1.38 (1–1.91)	0.99 (0.66–1.49)a
>75	1,983 (6.5)	1.87 (1.3–2.69)	1.98 (1.25–3.14)
Sex, %			
Male	9,881 (32.4)	1.14 (0.97–1.33)	1.35 (1.07–1.69)
BMI (kg/m²)			
30 to 34.9	10,681 (35.0)	–	–
35-39.9	7,337 (24.1)	1.05 (0.88–1.26)^a^	1.13 (0.86–1.49)^a^
>40	12,474 (40.9)	0.97 (0.81–1.17)^a^	0.97 (0.74–1.28)^a^
Stroke and CBVD			
No stroke or CBVD	28,861 (94.6)	Reference	Reference
Ischemic stroke	388 (1.3)	1.08 (0.65–1.8)^a^	1.87 (1.04–3.33)
Unspecified stroke or CBVD without stroke	1,243 (4.1)	0.99 (0.73–1.38)^a^	1.73 (1.18–2.54)
History of Cancer	4,170 (13.7)	1.60 (1.32–1.93)	0.97 (0.73–1.29)^a^
Anemia	4,282 (14.0)	1.02 (0.83–1.25)^a^	1.13 (0.86–1.5)^a^
CKD stage III	3,231 (10.6)	1.46 (1.2–1.78)	1.27 (0.94–1.71)^a^
COPD	2,431 (8.0)	0.98 (0.75–1.27)^a^	1.52 (1.09–2.11)
History of VTE	1,580 (5.2)	5.62 (4.71–6.7)	0.86 (0.52–1.40)^a^
History of non-major bleeding	4,074 (13.4)	1.01 (0.81–1.26)^a^	2.45 (1.92–3.12)
History of major bleeding	207 (0.7)	2 (1.13–3.55)	2.62 (1.29–5.32)
ACEi/ARB	8,501 (27.9)	0.96 (0.81–1.14)^a^	0.97 (0.75–1.24)^a^
Antiplatelets^b^	333 (1.1)	0.85 (0.45–1.62)^a^	2.39 (1.34–4.24)

Abbreviations: *HR* hazard ratio, *CI* confidence interval, *MB* major bleeding, *CBVD* cerebrovascular disease, *CKD* chronic kidney disease, *COPD* chronic obstructive pulmonary disease, *VTE* venous thromboembolism, *ACEi* angiotensin-converting enzyme inhibitors, *ARB* angiotensin II receptor blocker

^a^ not statistically significant at 5% level ^b^ Additional variables were analyzed in the full model, but not included in the final table as they were not statistically significant at 5% level and/or clinically meaningful. The list is as follows: thrombophilia, history of tobacco use, coronary heart disease, central venous catheter, history of diabetes, lower limb paralysis, hormone replacement therapy, major hematological and immunological conditions, drug misuse disorder, anticoagulants, peripheral vascular disease, severe varicosities, history of a heart failure comorbidity, statins, hypertension, HIV, coagulation disorder, moderate or severe chronic liver disease, calcium channel blockers, gastroduodenal ulcers, beta blockers,

## Discussion

Our study showed a non-negligible 90-day risk of VTE for patients with obesity in a surgical population (2.5%). Since there is a lack of specific guidelines’ recommendations on dosing and duration of pharmacological thromboprophylaxis in this patient population, a careful evaluation is warranted on whether prevalent practice patterns and recommended strategies are optimal for the reduction in the risk of VTE. The subgroup with the highest 90-day risk of VTE was thoracic surgery (4.9%). The highest net clinical value of VTE prophylaxis was in patients undergoing orthopedic surgeries, for whom high VTE and low MB risks were observed simultaneously. While we did not observe that VTE risk further increases with BMI among patients with obesity, similar to prior studies, we see a higher VTE risk among older patients with obesity and among patients with obesity who also have previous VTE history [4, 9, 26–29].

Results of this study add to the limited prior evidence investigating thromboprophylaxis in patients with obesity. Average thromboprophylactic daily dose of enoxaparin in our study was 70 mg, with the highest average dose of enoxaparin observed in patients with orthopedic surgery (Figure S4). Though there are potential concerns over higher bleeding risk related to higher dose thromboprophylaxis, several previous studies have demonstrated the advantages of adjusting thromboprophylactic dosage to a patient’s weight to achieve adequate VTE control [30–35]. Prior studies also concluded that a higher dose of thromboprophylaxis is safe and resulted in a significant reduction of VTE risk compared to standard thromboprophylaxis in patients with obesity [33, 36, 37].

The ESAIC guidelines on peri-operative VTE prophylaxis in patients with obesity recommend higher doses of LMWH, particularly in patients with BMI > 40 or weight > 150 kg. It also recommends extending pharmacological prophylaxis for at least 10 days rather than limiting it to hospital stays for patients at high risk of VTE, such as patients with obesity [38]. 

In our study, patients were followed for VTE and MB events for up to 90 days from the index, which is a longer follow up than seen in previous reports [10, 11]. We have observed that around 80% of VTE events occurred post-discharge and 40% occurred between 30 and 90 days of follow-up. In the National Surgical Quality Improvement Program (NSQIP) post-discharge VTE only accounted for about a third of VTE database because the follow-up is limited to 30 days [39]; however, this study and several previous reports showed a high post-discharge incidence of VTE in surgical patients and VTE risk persists up to 90 days after surgery and beyond the hospital stay [10, 20, 21]. Therefore, extending VTE prophylaxis after discharge might significantly decrease the proportion of post-discharge VTE events with subsequently associated decreases in morbidity and mortality.

A key strength of our study is the summary of characteristics and 90-day event rates from a large contemporary population. The study population was well classified in terms of clinical conditions and medications at baseline as the databases (electronic health records, claims, enrolment information, pharmacy, and labs) were linked. Linked database analysis offered high numbers of patients and covariates that increased the detection of post-discharge VTE and MB risk in patients. An extended and consistent follow-up period of 90 days was also taken to monitor VTE and MB, which evaluated the long-term postoperative VTE and MB risks following discharge from hospital in patients with obesity. We meticulously calibrated and enhanced the algorithm for the identification of VTE and MB over the methods used in other studies. Unlike most other studies which limit their population to patients with obesity who underwent bariatric surgery, our study assessed patients with obesity undergoing a broader range of surgeries. This analysis of patients in usual clinical practice may prove useful to multiple stakeholders, including payers, regulatory authorities, clinicians, and clinical guidelines committees for the purpose of identifying those at highest risk of VTE and MB despite being initiated on thromboprophylaxis.

As this study was based on observational data, several limitations should be noted. First, it only included individuals in the USA who were enrolled under a commercial or Medicare Advantage health plan, which may not be an accurate representation of non-insured populations- those covered under a public insurance program other than Medicare Advantage (e.g., Medicaid and other Medicare plans), or international populations. Second, in contrast to the highly stringent criteria used in RCTs, the current analysis relied on an algorithm based on codes and other information available in the database to identify VTE and MB endpoints; therefore, we cannot completely rule out the possibility of misclassification, for example due to the impact of mechanical prophylaxis. Our major bleeding definition, while informed by ISTH criteria for critical site bleeding, relies on diagnosis code position in claims data rather than validated clinical criteria. This approach cannot capture haemoglobin decline or transfusion requirements and may misclassify some non-critical bleeds (e.g., epistaxis requiring admission) as major bleeding events. These opposing biases may result in misestimation of major bleeding rates compared to studies using adjudicated ISTH criteria. Given that the entire population received enoxaparin, there is also the possibility of selection bias. In a similar vein, the database did not capture OTC medication, which could lead to an underestimation of antiplatelet use. This study did not look at death as an outcome as we did not link Optum data to the National Death Index; however, this is likely to be a minor limitation as fatal bleeds are expected to be rare. Our study may underascertain fatal pulmonary embolisms, which could be coded as sudden cardiac death, respiratory failure, or other non-specific causes of death. Additionally, fatal PEs occurring out-of-hospital or before diagnostic confirmation may not be captured in claims data. This could result in underestimation of true VTE rates and bias toward less severe presentations, potentially affecting risk-benefit assessments of thromboprophylaxis strategies. Finally, the risk factors in our study reflect patients’ status at index before follow-up; however, as the patient population represents hospitalized individuals, clinical characterization and risk factors could have changed over the course of hospitalization which are not accounted for in this study.

Despite being initiated on enoxaparin prophylaxis, patients experienced a clinically meaningful 90-day risk for VTE, at 2.5%, suggesting a need for further evaluation of recommended strategies for thromboprophylaxis with special emphasis on patients hospitalized due to thoracic surgery and those with a history of cancer. Patients with prior history of VTE are more prone to developing VTE during their follow-up period. Further, select patient subgroups seem to have a beneficial risk-benefit profile for extended or high dose prophylaxis, and formal cost-effectiveness analysis as well as risk-adjusted analyses or propensity score matching to account for baseline differences between groups would be beneficial to conduct. Risk stratification, such as using an augmented model with more clinical inputs based on these factors may help determine the optimal thromboprophylaxis strategy in surgical patients with obesity. Our findings support the need for individualized risk stratification to identify surgical patients with obesity who may benefit from extended prophylaxis beyond hospital discharge. While formal Caprini scoring requires clinical data beyond the scope of claims databases, our identification of key risk factors (thoracic surgery, history of VTE, cancer, age > 75) provides a foundation for clinical decision-making. Future prospective studies incorporating validated risk assessment tools such as the Caprini score would be valuable for developing evidence-based extended prophylaxis protocols in this population.

## Supplementary Information

Below is the link to the electronic supplementary material.

Supplementary Material 1 (DOCX 406 KB)

Supplementary Material 2 (PPTX 285 KB)

## Data Availability

No datasets were generated or analysed during the current study.
