# Publisher Correction: Venous Thromboembolism and Bleeding Risk in a Population with Obesity Hospitalized for Surgery and Receiving Enoxaparin for Thromboprophylaxis

**DOI:** 10.1007/s11695-026-08764-5

**Published:** 2026-05-29

**Authors:** Walter Ageno, Marc Carrier, Christine Stroh, Yasmina Djoudi, Mohamed Abdel-Moneim, Irfan Khan, Ekaterina Ponomareva, Juan I. Arcelus

**Affiliations:** 1 Department of Medicine, University of Padua, Padua, Italy; 2 Ottawa Hospital Research Institute, Ottawa Hospital, University of Ottawa, Ottawa, Canada; 3 Department of Obesity and Metabolic Surgery and Proctology, SRH Hospital Gera, Gera, Germany; 4 Sanofi (France), Paris, France; 5 Sanofi (UAE), Dubai, United Arab Emirates; 6 University of Sharjah, Sharjah, United Arab Emirates; 7 Sanofi (United States), Bridgewater, USA; 8 Axtria Inc., Berkeley Heights, USA; 9 Department of Surgery, University of Granada Medical School and Hospital Universitario Virgen de las Nieves, Granada, Spain

**Publisher Correction: Obesity Surgery**

10.1007/s11695-026-08606-4

Due to a production error the last named author and his affiliation are missing in the published article. The author is Juan I. Arcelus and his affiliation, #9, is 9 Department of Surgery, University of Granada Medical School and Hospital Universitario Virgen de las Nieves, Granada, Spain.

