# Supplementary material for: Venous Thromboembolism and Bleeding Risk in a Population with Obesity Hospitalized for Surgery and Receiving Enoxaparin for Thromboprophylaxis
Source: Obes Surg. 2026 May 18;36(7):3566–75. doi: 10.1007/s11695-026-08606-4 (PMC13323786; doi:10.1007/s11695-026-08606-4)
Supplement: Supplementary file 2 — Supplementary Material 2 (PPTX 285 KB) [file 11695_2026_8606_MOESM2_ESM.pptx]

## Slide 1
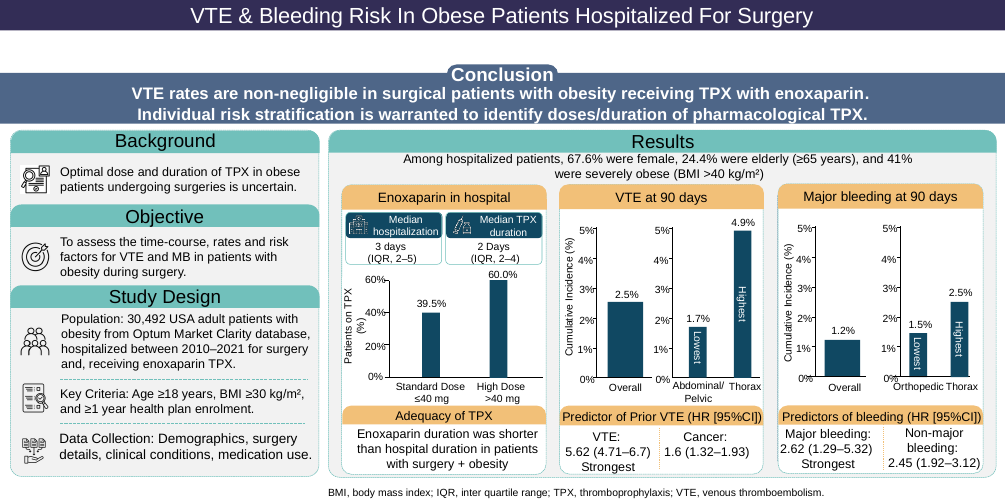

VTE & Bleeding Risk In Obese Patients Hospitalized For Surgery
Conclusion
VTE rates are non-negligible in surgical patients with obesity receiving TPX with enoxaparin.
Individual risk stratification is warranted to identify doses/duration of pharmacological TPX.
Results
Background
Among hospitalized patients, 67.6% were female, 24.4% were elderly (≥65 years), and 41%
were severely obese (BMI >40 kg/m²)
Optimal dose and duration of TPX in obese patients undergoing surgeries is uncertain.
Major bleeding at 90 days
VTE at 90 days
Enoxaparin in hospital
Objective
Median hospitalization
Median TPX duration
4.9%
5%
4%
3%
2%
1%
0%
5%
4%
3%
2%
1%
0%
5%
4%
3%
2%
1%
0%
5%
4%
3%
2%
1%
0%
To assess the time-course, rates and risk factors for VTE and MB in patients with obesity during surgery.
3 days
(IQR, 2–5)
2 Days
(IQR, 2–4)
60.0%
60%
Cumulative Incidence (%)
2.5%
Study Design
Cumulative Incidence (%)
2.5%
Highest
39.5%
Population: 30,492 USA adult patients with obesity from Optum Market Clarity database, hospitalized between 2010–2021 for surgery and, receiving enoxaparin TPX.
Patients on TPX (%)
40%
1.7%
1.5%
1.2%
Highest
20%
Lowest
Lowest
0%
Key Criteria: Age ≥18 years, BMI ≥30 kg/m², and ≥1 year health plan enrolment.
Abdominal/
Pelvic
Standard Dose
≤40 mg
High Dose
>40 mg
Thorax
Orthopedic
Thorax
Overall
Overall
Adequacy of TPX
Predictor of Prior VTE (HR [95%CI])
Predictors of bleeding (HR [95%CI])
Enoxaparin duration was shorter than hospital duration in patients with surgery + obesity
Data Collection: Demographics, surgery details, clinical conditions, medication use.
Non-major bleeding:
2.45 (1.92–3.12)
Major bleeding: 2.62 (1.29–5.32)
Strongest
VTE:
5.62 (4.71–6.7)
Strongest
Cancer:
1.6 (1.32–1.93)
United States
BMI, body mass index; IQR, inter quartile range; TPX, thromboprophylaxis; VTE, venous thromboembolism.
